# Novel Conductive Polymer Composite PEDOT:PSS/Bovine Serum Albumin for Microbial Bioelectrochemical Devices

**DOI:** 10.3390/s24030905

**Published:** 2024-01-30

**Authors:** Sergei E. Tarasov, Yulia V. Plekhanova, Aleksandr G. Bykov, Konstantin V. Kadison, Anastasia S. Medvedeva, Anatoly N. Reshetilov, Vyacheslav A. Arlyapov

**Affiliations:** 1 G.K. Skryabin Institute of Biochemistry and Physiology of Microorganisms, Pushchino Center for Biological Research of the Russian Academy of Sciences, 5 Prosp. Nauki, Pushchino, 142290 Moscow, Russia; setar25@gmail.com (S.E.T.); yu_plekhanova@pbcras.ru (Y.V.P.); agbykov@rambler.ru (A.G.B.); anatol@ibpm.pushchino.ru (A.N.R.); 2 Federal State Budgetary Educational Institution of Higher Education, Tula State University, 300012 Tula, Russia; kosya.kadison.032@mail.ru (K.V.K.);

**Keywords:** conductive composite, PEDOT:PSS, Nafion, bovine serum albumin, microbial biosensor, microbial fuel cell, thermally expanded graphite, *Gluconobacter oxydans*

## Abstract

A novel conductive composite based on PEDOT:PSS, BSA, and Nafion for effective immobilization of acetic acid bacteria on graphite electrodes as part of biosensors and microbial fuel cells has been proposed. It is shown that individual components in the composite do not have a significant negative effect on the catalytic activity of microorganisms during prolonged contact. The values of heterogeneous electron transport constants in the presence of two types of water-soluble mediators were calculated. The use of the composite as part of a microbial biosensor resulted in an electrode operating for more than 140 days. Additional modification of carbon electrodes with nanomaterial allowed to increase the sensitivity to glucose from 1.48 to 2.81 μA × mM^−1^ × cm^−2^ without affecting the affinity of bacterial enzyme complexes to the substrate. Cells in the presented composite, as part of a microbial fuel cell based on electrodes from thermally expanded graphite, retained the ability to generate electricity for more than 120 days using glucose solution as well as vegetable extract solutions as carbon sources. The obtained data expand the understanding of the composition of possible matrices for the immobilization of *Gluconobacter* bacteria and may be useful in the development of biosensors and biofuel cells.

## 1. Introduction

Bioelectrocatalytic systems such as microbial biosensors and biofuel cells are a rapidly expanding field of research that utilizes the metabolism of microorganisms to produce a usable electrical signal [1]. Electrochemical microbial biosensors are analytical platforms in which microorganisms are coupled to an electrode transducer for the rapid, accurate, and sensitive detection of target analytes [2]. These devices are actively used for environmental analysis—determination of BOD of aqueous media [3], total water toxicity [4], and levels of various substances in industrial waste, including food waste [5]. A microbial fuel cell (MFC) is an energy conversion device that utilizes electrogenic bacteria capable of producing electrical energy directly from organic waste while removing pollutants from the environment [6]. Waste from vegetable farms and food industry enterprises contains large amounts of polysaccharides, polyphenols, sugars, carotenoids, and glycosides. Prior to returning this waste to the environment, it must be tested to determine if it can be reused. Some of these compounds can be reused as animal feed, as precursors for the synthesis of valuable compounds, or as biofuel. Microbial biosensors can be used to determine the content of usable substances in such wastes, while MFCs can not only monitor the amount of useful compounds in food waste, but also effectively process them, simultaneously cleaning the environment and generating electricity. Numerous similar devices have been described in the literature that utilize various food wastes as an effective source of energy generation and bioremediation [7,8,9,10,11]; however, many aspects of the operations associated with such devices have not yet been fully explored.

One of the main tasks in the creation of microbial biosensors and MFCs is attaching the biocatalyst to the electrode surface while maintaining the activity and stability of the biocatalyst for a long time, as well as its secure adhesion to the electrode surface [12]. There are many polymers used to immobilize microorganisms on the surface of electrodes of biosensors and biofuel cells, allowing them to maintain their activity for a long time [13,14,15]. The use of polymer gels for the immobilization of bacterial cells and enzymes has some advantages over other methods, since it allows the immobilization of cells in a more effective and simpler way, reliably protecting them from the external environment and ensuring strong attachment to the surface of the working electrode [16]. Polymers used for the immobilization of biocatalysts on conductive electrodes can be divided into two groups—natural and synthetic. Natural polymers include polysaccharides, alginic acid salts, carrageenans, chitin and chitosan, gelatin, agar, and protein hydrogels. Synthetic matrices for immobilization include poly(vinyl alcohol), polyacrylamide, and polyethylene glycol. For the most part, these polymers are not highly conductive, which requires their additional modification with nanomaterials and limits their use in electrochemical devices [17].

One of the promising conducting polymers is poly(3,4-ethylenedioxythiophene): poly(styrenesulfonate) (PEDOT:PSS), which has a mixed electron-ionic type of conductivity [18]. Electron transfer in the PEDOT polymer occurs through a system of conjugated bonds due to electron exchange reactions between neighboring redox sites, and is also accompanied by the conjugate movement of dopant anions along the polymer chain [19]. This allows it to be used to create a conductive matrix that ensures the transfer of electrons from the enzyme systems of microorganisms to the surface of the working electrode. The application of PEDOT:PSS results in MFC anodes with high stability and easy modification [20,21,22]. It is worth noting that in some cases, PEDOT:PSS has a negative effect on bacterial cells [23]. Researchers are constantly looking for new ways to improve the conductive properties of PEDOT-based films. However, the stability of PEDOT films still remains a major challenge for practical measurements. One of the options for improving physical properties of PEDOT-conductive films is to dope them with additional compounds. Bovine serum albumin (BSA) can be used as one of these stabilizing agents. BSA contains many charged amino groups and can interact with the surface of carbon electrodes due to hydrophobic force, hydrogen bonds, van der Waals, and π stacking interactions [24]. In addition, the incorporation of BSA can enhance the adhesive properties of PEDOT:PSS and stabilize the conductive composition.

There are research articles in which BSA is used as a template for the electrochemical synthesis of PEDOT on platinum electrodes [25]. In this case, a PEDOT:BSA composite is considered, in which the protein replaces PSS, which is a standard stabilizer of PEDOT chains. The combined use of BSA and PSS in a single composite is not well studied. Researchers [26,27] have studied the physical parameters of the BSA-PEDOT:PSS composition and their changes when glucose was included in its composition, but not the stability of this composition when used as an immobilization matrix. We were not able to find any papers describing the use of this composition for the immobilization of microbial cells in biosensors and biofuel cells.

## 2. Materials and Methods

### 2.1. Reagents

Monopotassium phosphate, sodium hydroxide, sodium chloride, anhydrous acetic acid, d-glucose, glutaraldehyde (Mosreaktiv, Moscow, Russia); bovine serum albumin (fraction V, protease-free) (ICN Biomedicals Inc., Maumee, OH, USA), 2,6-dichlorophenolindophenol sodium salt, chitosan (low molecular weight), Nafion 117 (5% solution), PEDOT:PSS (1.3 wt % dispersion in H_2_O), bacteriological agar, potassium ferricyanide (Merck, Burlington, MA, USA); ethanol, sorbitol, yeast extract, and potassium chloride (Dia-M, Moscow, Russia) were used. Three-contact screen-printed carbon electrodes were purchased from Color Electronics (Moscow, Russia) and were used as electrodes in biosensors. The working electrode was 3 mm in diameter and was surrounded by a graphite auxiliary electrode and a silver reference electrode. Thermally expanded graphite (TEG) synthesized as described in [28] was used as a basis for the working electrodes of MFCs.

### 2.2. Microorganism Cultivation

The *Gluconobacter oxydans* sbsp. *industrius* VKM B-1280 (All-Russian Collection of Microorganisms) strain was used as the bioreceptor. Cells were grown according to the method described in [29]. After the cultivation, the *G. oxydans* cell suspension was washed with phosphate buffer and diluted to a concentration of 1 mg wet weight per µL. This suspension was further used to form the bioreceptors.

### 2.3. Biosensor Preparation

To create a conductive composition on the surface of the working electrode, a mixture of PEDOT:PSS polymer and BSA (1 or 7% aqueous solution) in various volume ratios was used. The resulting suspension was mixed using a Biosan V-32 multi-vortex for 30 s and applied to the working electrode (5 μL), air dried for 1 h at room temperature, and then for 12 h at +4 °C. To firmly immobilize bacterial cells, additional reagents were used, such as Nafion 117, chitosan (2% solution in 1% acetic acid), or glutaraldehyde (2.5%). Options for applying polymers, their quantity, and mixing sequence varied; data are presented in the Supplementary Materials (Figure S1). The mixture of *G. oxydans* cells/Nafion (5:2 *v*/*v*) was chosen as the most optimal option for biocatalyst immobilization. A schematic diagram of bioelectrode preparation is shown in Figure S2. The mixture in an amount of 5 μL was applied to the surface of the electrode and dried at room temperature for an hour. The number of cells on the electrode surface was 0.5 mg/mm^2^. Carbon nanomaterial, TEG, was used as an option to modify the biosensor electrodes. A layer of TEG with a thickness of 0.1 mm and a diameter of 3 mm was preliminarily fixed on the surface of the working electrode under a pressure of 150 bar. Biosensors were stored dry at 4 °C between measurements.

### 2.4. Instrumentation

All electrochemical measurements were conducted using a VersaStat 4 galvanostat potentiostat with the FRA module (Ametek, Berwyn, PA, USA) and an EmStat 3 galvanostat potentiostat (Palmsens, Houten, The Netherlands). All chronoamperometric measurements were carried out at an applied potential of +200 mV vs. Ag/AgCl electrode in the presence of a 2,6-dichlorophenolindophenol mediator (DCPIP) or potassium hexacyanoferrate (III) (HCF) in a glass vessel in a 25 mM potassium phosphate buffer (PBS), pH 6.0, containing 10 mM sodium chloride. Measurements were carried out at 25 °C at constant stirring (500 rpm). Cyclic voltammograms (CVs) were registered at a various scan rates within the range from −500 up to +500 mV. Impedance characteristics were measured at an applied potential of +200 mV vs. Ag/AgCl within the range of frequencies from 40 kHz up to 0.2 Hz at a voltage modulation amplitude of 10 mV in the presence of 5 mM potassium hexacyanoferrate (III).

### 2.5. MFC Characterization

In MFC prototypes, pressed sheets of thermally expanded graphite with an area of 40 mm^2^ were used as anodes and cathodes. To modify the anode, the composition of PEDOT:PSS/BSA/*G. oxydans*/Nafion was used. A dual chambered MFC consisted of two interconnected cuvettes, each with a working volume of 5 mL. The chambers were separated by an MF-4SK proton-selective membrane (Plastpolimer, St. Petersburg, Russia) with an area of 1.2 cm^2^. A 25 mM PBS, pH 6.5, containing 10 mM sodium chloride, was used as anolyte and catholyte; 2,6-Dichlorophenolindophenol (DCPIP, 0.14 mM) in the anode chamber and potassium hexacyanoferrate (III) (4 mM) in the cathode chamber were used as redox mediators. As carbon sources for *G. oxydans* cells, we used glucose at concentrations of 10 mM, as well as extracts obtained from beet and carrot waste (BOD was 8500 ± 1300 and 12,000 ± 1800 mg O_2_/dm^3^, respectively). Bioanodes were stored dry at 4 °C between measurements.

The MFC power characteristics and internal resistances were calculated using the formulas from [30].

### 2.6. Clark-Type Oxygen Electrode Measurements

Cell suspensions and mixtures of cells with the following polymers were prepared: Nafion, BSA, or PEDOT:PSS. A suspension or mixture was applied onto a fragment of Whatman GF/A (glass microfibre paper, UK), 3 × 3 mm in size, which was used as a bioreceptor for a Clark-type oxygen electrode. The amount of cell per electrode was 1 mg wet weight. The bioreceptor was dried for 15–25 min at room temperature and fixed on the surface of a Clark-type oxygen electrode. The measurements were carried out in a PBS (25 mM, pH 6.5) in a 2 mL acrylic vessel at constant stirring. The maximum rate of change in the output signal dI/dt (nA/s) was measured, related proportionally to the rate of change in the concentration of oxygen consumed by immobilized bioreceptor cells in response to the addition of a substrate—glucose, 0.5 mM. The measurements were conducted using an IPC-2L potentiostat (Kronas, Russia). The receptor was stored dry at a temperature of +4 °C between measurement.

### 2.7. Scanning Electron Microscopy (SEM)

The electron microscopic analysis of the samples was carried out using a Hitachi TM4000Plus scanning electron microscope (Hitachi, Tokyo, Japan) in a low-vacuum (30 Pa) mode during registration of secondary electrons.

## 3. Results and Discussion

**Figure 1:**
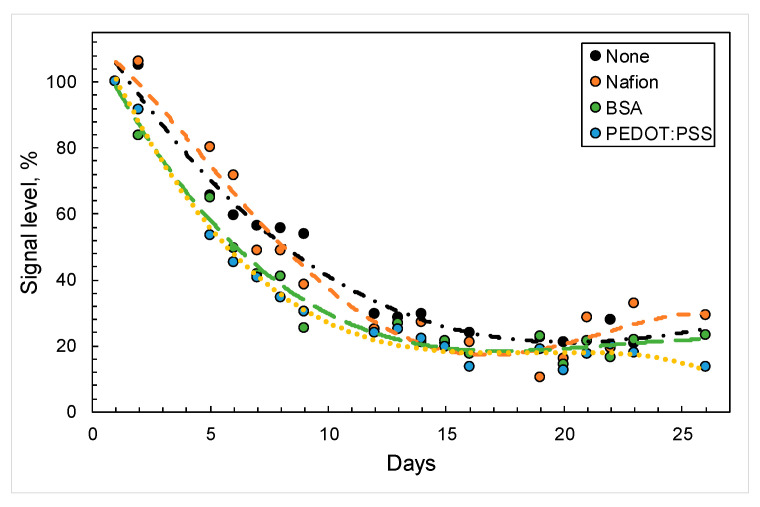
Changes in signals from biosensors based on *G. oxydans* cells in the presence of various compounds (black—no polymers; orange—Nafion; green—BSA; blue—PEDOT:PSS) upon the introduction of glucose (0.5 mM).

The use of conductive polymers often improves the parameters of both electrochemical biosensors and MFCs, but at the same time they can reduce the metabolic activity of bacteria during prolonged contact [31]. A biosensor based on a Clark-type electrode was used to evaluate the effect of the polymers utilized in the work on *Gluconobacter* cells. We assessed the effects of polymers that were in prolonged contact with bacterial cells on microbial respiratory activity during the utilization of the substrate. Figure 1 shows changes in biosensor responses after glucose injection over a month. It was shown that the drop in signal level was due to the natural decline in cell activity over time, and not caused by contact with the reagents under study. Therefore, we can conclude that the use of these compounds as part of electrochemical biosensors and MFCs should not have a negative effect on the activity of bacteria during long-term operation as part of the devices under study.

### 3.1. Assessment of Bioelectrochemical Properties of Bacteria in the Composite Composition

**Figure 2:**
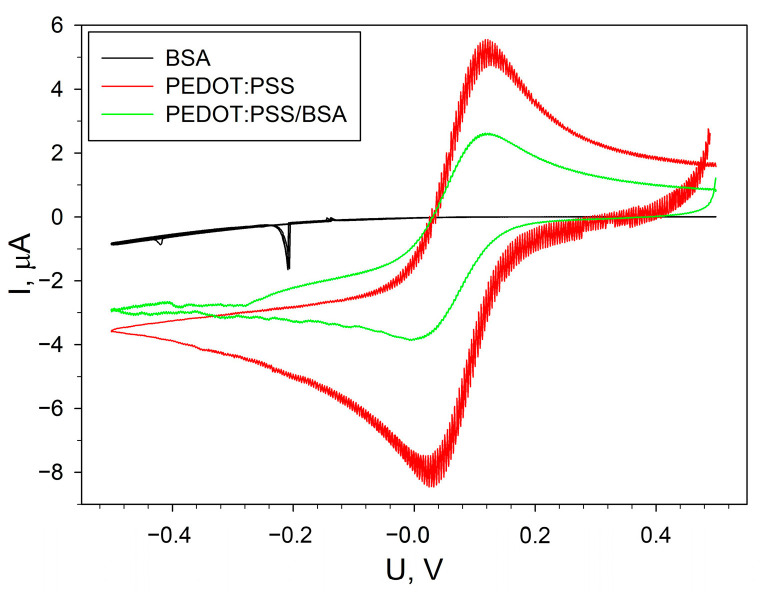
Cyclic voltammograms of electrodes modified with PEDOT:PSS, BSA, and PEDOT:PSS/BSA in the presence of 4 mM potassium hexacyanoferrate.

To assess the presence of electron transfer in the composition used for cell immobilization, the method of cyclic voltammetry was used. The electrode was modified with PEDOT:PSS or BSA separately, as well as with a mixture of them in a 1:1 ratio. To characterize the synthesized materials, Energy Dispersive X-ray (EDX) microanalysis was used (Table S1). Cyclic voltammograms were measured in the presence of potassium hexacyanoferrate (III) as a standard redox label [32]. The obtained voltammograms are presented in Figure 2. The electrode modified with BSA has no visible oxidation–reduction peaks of the mediator, which indicates the non-conducting nature of this material. The most pronounced redox peaks were observed when using PEDOT:PSS gel. However, this gel itself does not have suitable mechanical properties and is easily washed off from the carbon electrode surface. When adding even a small amount of BSA, the mechanical properties of the conductive composite are improved, while its redox activity is maintained.

**Figure 3:**
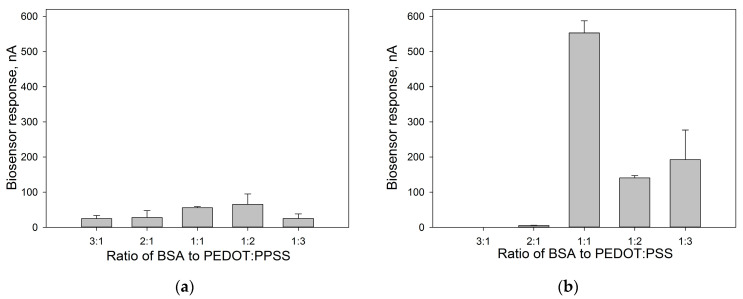
Amperometric responses of biosensors with various PEDOT:BSA ratios upon the introduction of 3 mM glucose when applying a conductive matrix in the form of a mixture (**a**) and when applying layer-by-layer components of the composite (**b**).

To immobilize bacterial cells, it is necessary to select the optimal ratio and order of application of the immobilizing agents. The method of applying the composite (mixture or layer-by-layer) was varied, and various additional compounds were tested that could improve cell adsorption on the electrode surface. Chitosan, Nafion, and glutaraldehyde were used as additional reagents. The most promising composition turned out to be PEDOT:PSS/BSA/*G. oxydans*/Nafion with a layer-by-layer application of the mixture components (Figure S1). For this composition, microphotographs were obtained by layer-by-layer application and by mixing all components on the surface of the working electrode (Figure S3). An important parameter is the ratio of PEDOT:PSS and BSA gels in the applied composite. We varied the ratios of PEDOT:PSS and BSA in the composite with a layer-by-layer and simultaneous application of all components and compared the chronoamperometric signals of the microbial biosensor upon the introduction of glucose. The amperometric responses of biosensors based on each composition are shown in Figure 3.

According to the data presented, the responses of biosensors made using the layer-by-layer method of applying components (Figure 3b) are several orders of magnitude higher than when using a mixture. The maximum amperometric signal was observed when using a mixture of PEDOT:PSS and BSA in a volume ratio of 1:1.

**Figure 4:**
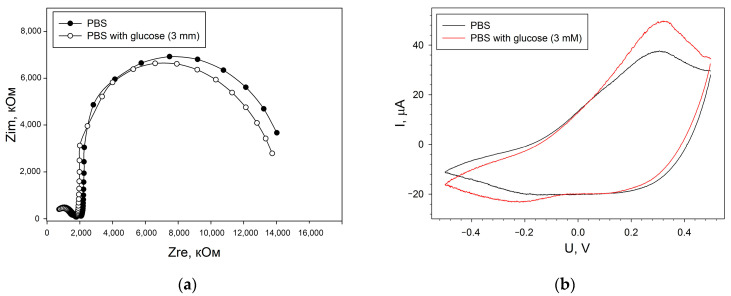
Changes in the electrochemical characteristics of biosensor electrodes when glucose (3 mM) is added to the measuring cell. (**a**)—Nyquist diagrams, (**b**)—cyclic voltammograms. Presented data are for electrode with PEDOT:PSS and BSA ratio of 1:1.

The compositions were also studied by electrochemical impedance spectroscopy. The data were obtained by adding 0.14 mM 2,6-DCPIP as a redox label, since 2,6-DCPIP is often used with *G. oxydans* cells [33]. For each composition, Nyquist diagrams were plotted in the presence and absence of glucose (Nyquist diagrams for electrodes obtained by applying a conductive matrix in the form of a mixture are presented in Figure S4). The resulting Nyquist plots were processed using a modified Randles equivalent circuit (Figure S4c). The parameter values of the studied layer-by-layer bioelectrodes are presented in Table 1. As can be seen from the data obtained, the lowest charge transfer resistance and the minimum total internal resistance of the electrode were observed for the composition with a PEDOT:PSS and BSA ratio of 1:1. It should be noted that for all studied compositions, the level of charge transfer resistance decreased when glucose (3 mM) was introduced into the measurement cell (Figure 4a). The transfer of electrons in the system upon the addition of a substrate was also observed using cyclic voltammetry (Figure 4b). The CV shows an increase in the oxidation current in the potential range from 0.1 to 0.5 V. This fact indicates that there is electron transfer in the system, which is affected by the oxidation of the substrate by bacteria. At the same time, Nyquist diagrams (Figure 4a) show a decrease in the diameter of a typical semicircle when adding a substrate, which is associated with a decrease in the charge transfer resistance in the system due to the transformation of the substrate by cells.

### 3.2. Evaluation of Heterogeneous Electron Transfer

When designing electrochemical devices, it is very important to select an effective electron carrier from the immobilized biological component to the electrode surface. Two soluble mediators were compared: 2,6-DCPIP, as the most commonly used mediator in combination with acetic acid bacteria, and HCF, as the most common soluble redox mediator. A study was carried out on the effect of the used mediators on the respiratory activity of bacteria using an oxygen electrode (data presented in Supplementary (Figure S5). It was shown that in the presence of HCF, the signal upon the addition of glucose decreases depending on the concentration by 32.5–45.1%, and in the presence of 2,6-DCPIP, depending on the concentration by 31.0–59.1%. After removing the mediator from the measurement cell, the signal for glucose does not return to its original value, decreasing by 13.9% in the case of using 2,6-DCPIP, and by 21% when using HCF. Therefore, 2,6-DCPIP is considered to be less toxic to the bacterial cells used in our study.

**Figure 5:**
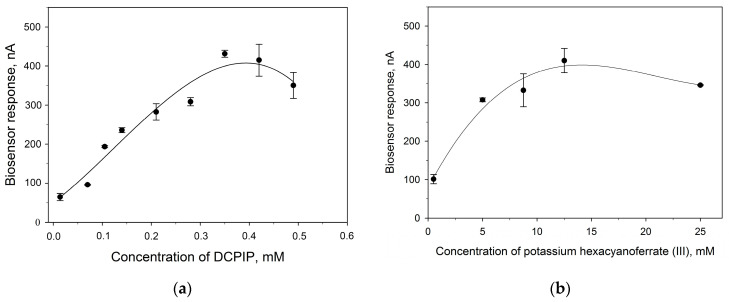
Amperometric signals of biosensors vs. the concentration of redox mediators used: (**a**)—2,6-DCPIP, (**b**)—HCF.

Figure 5 shows the dependences of the responses of amperometric biosensors on the concentration of redox mediators upon the addition of 3 mM glucose. From the presented data, it is clear that the maximum signal level for the two mediators is nearly identical, which amounts to about 400 nA. To assess the efficiency of the electron transfer of two redox pairs, the heterogeneous rate constants of electron transfer to the electrode were calculated. In the “bacteria–mediator–electrode” system, electron transfer occurs in several stages. First, the redox mediator diffuses to the electrode surface, then the mediator is adsorbed on the electrode surface, electron transfer to the electrode, desorption of the reaction product, and its diffusion [34]. The rate of the process is determined by the slowest stage. To identify this stage, as well as to calculate the kinetic constants of electron transfer, the method of cyclic voltammetry was used. We obtained cyclic voltammograms of bioelectrodes at different potential scan rates in the presence of 2,6-DCPIP and HCF, as well as the dependences of anodic and cathodic currents on the scan rate (Figure S6). Both cathodic and anodic peak potentials shifted in positive and negative directions with increasing scanning speed. At the same time, the levels of redox couple currents increased gradually with increasing scanning speed. The linearity of the dependence of currents on the scanning speed confirms that the processes occurring on the electrodes are quasi-reversible, and the limiting stage is the transfer of an electron from the biological component on the surface of the electrode, and not the diffusion of the substrate from the solution to the active centers of the enzyme.

To estimate the rate of the heterogeneous electron transfer process, the value of the constant k_s_ was calculated using the Laviron equation [35]:

(1) $$Lnk_{s}=\alpha Ln\left(1-\alpha \right)+\left(1-\alpha \right)Ln\alpha -Ln\frac{RT}{nFv}-(1-\alpha )\frac{nF{∆E}_{p}}{RT},$$

where ∆E, the potential difference between the anodic and cathodic peaks, υ is the scan speed, n is the number of electrons, α is the charge transfer coefficient, R is the gas constant, T is temperature, and F is Faraday’s constant.

The value of the α × n can be calculated from the slope of the dependence of the anode peak on the scanning speed. In addition, changes in heterogeneous transfer rates were checked when the working electrode was modified with a carbon nanomaterial, thermally expanded graphite. The obtained values of k_s_ for various electrodes and redox mediators are presented in Table 2. Based on the presented values, the value of k_s_ when using DCPIP is higher than when using HCF. This indicates the advantage of using DCPIP with acetic acid bacteria in biosensors. In addition, modification of the surface of the working electrode with TEG also leads to an increase in the rate of heterogeneous transfer in the system. Thus, the use of carbon nanomaterial reduces the resistance in the system and facilitates electronic transport.

### 3.3. Analytical Characteristics of Biosensors

The analytical characteristics of an amperometric biosensor based on the PEDOT:PSS/BSA/*G. oxydans*/Nafion composition were studied. First, the operational stability and long-term storage stability of the biosensor were determined. The developed microbial biosensor provides a stable signal, since the relative standard deviation did not exceed 10% (Figure S7), and the biosensor remained usable for more than 4 months. The level of the biosensor signal upon the introduction of 3 mM glucose after 140 days of storage was 23% of the initial level. Changes in the amplitude of signals from amperometric biosensors during storage are shown in Figure S8.

**Figure 6:**
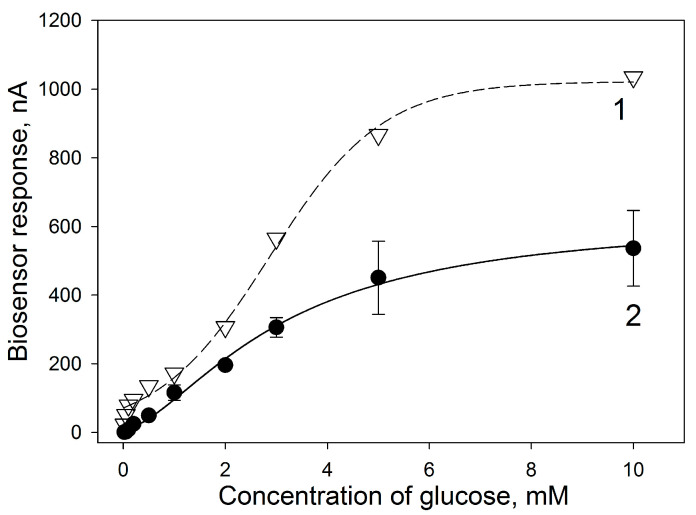
Calibration curves for PEDOT:PSS/BSA/*G. oxydans*/Nafion electrodes modified (1) and not modified with TEG nanomaterial (2).

The calibration curves for the microbial biosensor modified with thermally expanded graphite and not modified with nanomaterial are presented in Figure 6.

The detection range of both biosensors was 0.02–10 mM of glucose. The calibration curves were fit by the three-parameter Hill equation (R^2^ = 0.98):

(2) $$I=\frac{I_{max}\times S^{h}}{K_{M}^{h}+S^{h}},$$

where I_max_ is the maximum biosensor signal achieved when the substrate concentration (S) tends to infinity; $K_{M}^{h}$ is the apparent Michaelis–Menten constant; h is the Hill coefficient.

The values of the biosensors’ parameters are presented in Table 3.

As can be seen from the presented data, modification of the bioelectrode with a nanomaterial leads to an increase in the sensitivity of the biosensor to glucose by approximately two times (from 1.48 to 2.81 μA × mM^−1^ × cm^−2^), as well as an increase in the value of the Hill coefficient from 1.6 to 2.4. The Hill coefficient (h) is a dimensionless quantity that characterizes the cooperativity of ligand binding by an enzyme. In both cases, the value of h is greater than one, that is, positive cooperativity of enzymes is observed for immobilized bacterial cells. Since the kinetics of the enzymatic reaction is described by the Hill equation, this confirms that the enzyme requires a cofactor (flavin adenine dinucleotide) for the reaction of substrate oxidation. The cofactor forms the active center together with the functional groups of amino acid residues of the enzyme, where binding to the substrate occurs and an activated enzyme–substrate complex is formed. An increase in the value of Hill coefficient in the presence of TEG shows that modification with a nanomaterial facilitates these processes. The Michaelis constant shows the degree of affinity between the substrate and the enzyme—the lower the value of the constant, the higher the affinity of the enzyme for the substrate. The affinity of the enzymes for the substrate remained virtually unchanged when TEG is introduced into the system. Thus, modification of the bioelectrode by introducing TEG does not affect the metabolic processes in cells, but increases the conductivity between the polymer composite and the electrode, which leads to an increase in the maximum current level in the system and its sensitivity. Since increasing the maximum current level improves the efficiency of bioelectrochemical devices, it may be useful in the development of microbial fuel cells. Therefore, the further developed composite was used to immobilize *G. oxydans* bacterial cells in MFC based on electrodes made entirely of thermally expanded graphite.

### 3.4. Practical Application of Composite as a Basis for an MFC

**Figure 7:**
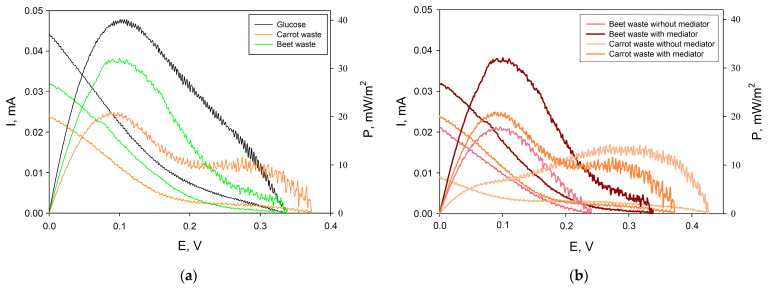
Polarization curves obtained from cyclic voltammograms and power density curves for MFC operating on three different substrates. The mediator HCF (5 mM) was always present in the cathodic compartment. In the anodic compartment, the mediator 2,6-DCPIP (0.14 mM) was either present (**a**) or absent (**b**).

Several prototypes of a microbial fuel cell based on the studied composites were constructed. The electrodes were made of pressed TEG plates. The polarization and power density curves recorded for the MFC prototypes are shown in Figure 7. Glucose, as well as extracts from beet and carrot waste, were used as a carbon source for the operation of the MFC. The amount of power generated by MFC increases in the series, carrots < beetroot < glucose (10 mM), and reaches a value of 40 mW/m^2^ for the glucose model solution. It is worth noting that carbohydrate residues are present in both beet and carrot waste, which allows them to be used as a carbon source for the continuous operation of MFC. In addition, as shown in Figure 7b, power in solutions of beet and carrot waste is generated without introducing an electron transport mediator into the anode compartment of the MFC. This can be explained by the fact that vegetable waste solutions may contain redox-active compounds (for example, caffeic acid, ferulic acid, etc.) [36], which facilitate electron transport between microbial cells and the electrode surface. Thus, the created MFC models can be used to clean agricultural wastewater without adding additional reagents that may be toxic to the environment.

**Figure 8:**
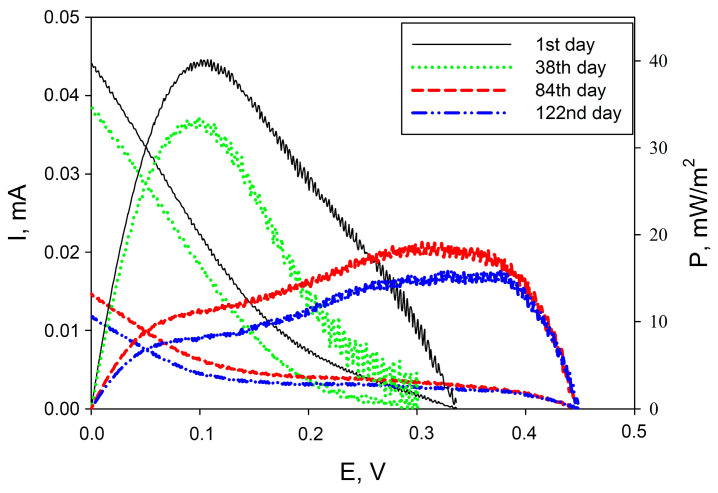
Changing polarization curves obtained from cyclic voltammograms and power density curves for MFC during its operation. MFC prototypes were stored dry at +4 °C between measurements.

The studied composite was capable of maintaining the activity of bacterial cells on the surface of TEG electrodes for more than 120 days. Figure 8 shows examples of changes in the polarization and power dependences of MFC. During the first month of MFC operation, the power drop was only 17.5%. The MFC power decreased from 40 to 17 mW/m^2^ on the 84th day of cell operation. Thus, the proposed composite not only improves the conductivity of the system and the rate of electron transfer in it, but also provides a suitable microenvironment for the long-term operation of acetic acid bacteria. The data obtained were compared with the parameters of other MFCs presented in the literature (Table 4).

The maximum voltage and the power density obtained in this work are comparable with similar parameters for the devices presented in the recent papers. It is worth noting that compared to the PEDOT:PSS/Graphene/Nafion combination [43], the composition presented in this work provides slightly lower power (due to the absence of graphene), but still allows the MFC to generate electricity for a longer period of time.

## 4. Conclusions

This paper presents a new conductive polymer composite based on PEDOT:PSS, BSA, and Nafion, which was used for the effective immobilization of acetic acid bacteria on graphite electrodes as part of microbial biosensor and biofuel cell prototypes. It has been shown that individual components of the composite do not have a significant negative effect on the catalytic activity of microorganisms. The developed microbial biosensor was capable of providing a measurable signal for more than 140 days. When used as part of MFC, the cells retained the ability to generate electricity for more than 120 days. The additional modification of graphite electrodes with thermally expanded graphite allowed us to increase the level of biosensor amperometric signals by approximately two times and its sensitivity from 1.48 to 2.81 μA × mM^−1^ × cm^−2^, without affecting the affinity of bacterial enzyme complexes for substrates. A prototype MFC based on electrodes made of thermally expanded graphite generated power at the level of 40 mW/m^2^ when using a model glucose solution, as well as vegetable waste solutions. In addition, the possibility of generating electricity from microbial MFC in vegetable waste solutions without introducing additional redox compounds has been shown. This composition was used for the immobilization of bacterial cells as part of bioelectrochemical devices for the first time, and test experiments were carried out on the use of MFC based on this composition for processing agricultural waste.

## Figures and Tables

**Table 1:** Electrochemical parameters of bioelectrodes obtained using the electrochemical impedance spectroscopy method.

Ratio of PEDOT:PSS and BSA in the Composition	R_S_ *, Ohm	С **, F	R_ct_ ***, Ohm
3:1	2700	2.33 × 10^−8^	7.97 × 10^7^
3:1 с with glucose	2354	2.36 × 10^−8^	6.26 × 10^7^
2:1	1555	1.23 × 10^−8^	3.71 × 10^7^
2:1 with glucose	1359	1.73 × 10^−8^	2.16 × 10^8^
1:1	582	1.33 × 10^−6^	1.20 × 10^4^
1:1 with glucose	322	5.20 × 10^−5^	1.16 × 10^4^
1:2	534	4.40×10^−5^	1.12 × 10^6^
1:2 with glucose	512	4.50 × 10^−5^	2.28 × 10^5^
1:3	480	6.60 × 10^−5^	3.73 × 10^5^
1:3 with glucose	471	7.20 × 10^−5^	1.31 × 10^5^

* Rs—electrolyte resistance, ** C—capacity of the electrical double layer, *** R_CT_—resistance of charge transfer.

**Table 2:** Rate constants of heterogeneous electron transfer to a screen-printed electrode for two redox mediators.

Electrode Modification	Mediator	n	a	k_s_
TEG/PEDOT:PSS/BSA/*G. oxydans*/Nafion	DCPIP	2	0.0240	0.0168
TEG/PEDOT:PSS/BSA/*G. oxydans*/Nafion	HCF	1	0.0164	0.0065
PEDOT:PSS/BSA/*G. oxydans*/Nafion	DCPIP	2	0.0247	0.0152
PEDOT:PSS/BSA/*G. oxydans*/Nafion	HCF	1	0.0097	0.0038

**Table 3:** Values of analytical parameters of biosensors calculated from the Hill equation.

Composite	Printed Electrode Not Modified by TEG	Printed Electrode Modified by TEG
Parameter		
Hill coefficient	1.60 ± 0.38	2.41 ± 0.38
Apparent Michaelis constant	3.08 ± 0.61	3.13 ± 0.25
Biosensor sensitivity, µA × mM ^−1^ × cm^−2^	1.48	2.81
Linear detection range, mmol	0.25–3.76	0.57–4.62
Detection range, mmol	0.02–10	0.02–10
Minimum detectable concentration, mmol	0.02	0.02
I_max_, nА	661	1035

**Table 4:** Comparison of performances of different MFCs presented in the literature.

Electrode Material	Anode Modification	Biocatalyst	Carbon Source	Maximum Voltage, mV	Power Density, mW/m^2^	Reference
Carbon cloth	Au@polyaniline	*Escherichia coli*	Glucose	640	804	[37]
Biochar	PEDOT/NiFe_2_O_4_	Microbial consortium	Glucose	690	1200	[38]
Carbon felt	-	*Shewanella baltica*	Artificial wastewater	190	12	[39]
Graphite rod	-	Microbial consortium	Potato waste	112	current density—36.84 mA/m^2^	[7]
Graphite rod	-	Microbial consortium	Fruit waste	180	0.71	[8]
Carbon felt	oxidation	*Rhizobium anhuiense*	Glucose	683	4.93	[40]
Graphite felt cloth	heated at 110 °C	Microbial soil consortium	Vegetable waste	305	89	[41]
Copper	-	Microbial consortium	Lettuce waste	959	378	[42]
Carbon veil	PEDOT:PSS	Microbial consortium	Human urine	640	61	[21]
Graphite rod	PEDOT:PSS/Graphene/Nafion	*Gluconobacter oxydans*	Municipal wastewater	480	65	[43]
Graphite felt	MWCNT/polyaniline	Microbial consortium	Cauliflower leaf waste	681	10.1 (W/m^3^)	[44]
Thermally expanded graphite	PEDOT:PSS/BSA/Nafion	*Gluconobacter oxydans*	Vegetable waste	440	40	This study

## Data Availability

The original contributions presented in the study are included in the article/Supplementary Material, further inquiries can be directed to the corresponding author.

## Supplementary Materials

The following supporting information can be downloaded at: https://www.mdpi.com/article/10.3390/s24030905/s1, Figure S1: Dependence of biosensor signals on the composition of the working electrode; Figure S2: A schematic diagram of bioelectrode preparation and electron transport in system; Figure S3: Scanning electron micrographs of the surface of a graphite electrode covered with composition no. 1 (a) and composition no. 8 (b); Figure S4: Nyquist diagrams for electrodes with different compositions of the working electrode: (a) in the absence of glucose, (b) in the presence of 3 mM glucose. The ratio of PEDOT:PSS and BSA is shown. (c)—Randles scheme; Rs—electrolyte resistance, C—electrode capacity, Rct—electrode charge transfer resistance, Zw—Warburg element; Figure S5: The influence of electron transport mediators on the respiratory activity of cells in the presence of a substrate (glucose, 0.5 mM). (a) with the addition of HCF and (b) 2,6-DCPIP; Figure S6: Electrochemical characteristics of the electrode based on the PEDOT:PSS/BSA/*G. oxydans*/Nafion composition in the presence of redox mediators: 1—DCPIP; 2—HCF. (a)—cyclic voltammograms of electrodes at different scan rates; (b)—linear dependences of the anodic and cathodic peak currents on the scanning speed (I_pa_—the magnitude of the anodic peak, I_pc_—the magnitude of the cathodic peak); (c)—linear dependences of the anodic and cathodic peak potentials on the natural logarithm of the scanning speed (E_pa_—anodic peak potential, E_pc_—cathodic peak potential); Figure S7: Operational stability of the electrode based on the conductive matrix PEDOT:BSA (1:1); Figure S8: Changes in biosensor signals during storage for 4.5 months. The dry biosensor was stored at +4 °C between measurements; Table S1: Elemental composition of bioreceptor components.
