# Supplementary material for: Novel Conductive Polymer Composite PEDOT:PSS/Bovine Serum Albumin for Microbial Bioelectrochemical Devices
Source: Sensors (Basel). 2024 Jan 30;24(3):905. doi: 10.3390/s24030905 (PMC10857495; doi:10.3390/s24030905)
Supplement: Supplementary file 1 [file sensors-24-00905-s001.zip › sensors-2811838-supplementary.pdf]

sensors

MDPI

Supplementary

Novel Conductive Polymer Composite PEDOT:PSS/Bovine Serum Albumin for Microbial Biolectrochemical Devices

Sergei E. Tarasov [1], Yulia V. Plekhanova [1], Aleksandr G. Bykov [1], Konstantin V. Kadison[2], Anastasia S. Medvedeva[2], Anatoly N. Reshetilov [1], and Vyacheslav A. Arlyapov [2,*]

[1] Federal Research Center «Pushchino Scientific Center for Biological Research of the Russian Academy of Sciences», G.K. Skryabin Institute of Biochemistry and Physiology of Microorganisms, Russian Academy of Sciences, 142290 Pushchino, Russia; setar25@gmail.com (S.E.T.); yu_plekhanova@pbcras.ru (Y.V.P.); agbykov@rambler.ru (A.G.B.); anatol@ibpm.pushchino.ru (A.N.R.)

[2] Federal State Budgetary Educational Institution of Higher Education, Tula State University, 300012 Tula, Russia; kosya.kadison.032@mail.ru (K.V.K.)

* Correspondence: v.a.arlyapov@tsu.tula.ru

Materials and Methods

Formation of a conductive layer of a bioreceptor on the surface of a graphite electrode. The number of *G. oxydans* cells was 0.5 mg/mm^2 on the surface of all electrode variations.

1. A mixture of PEDOT:PSS and BSA polymer (7% aqueous solution) in a volume ratio of 1:1 was applied to the working electrode in an amount of 5 µl, air-dried for 1 hour at room temperature, and then at + 4°C for 12 hours. Then a mixture of bacterial cells and Nafion (5:2 v/v) in an amount of 5 µl was applied on top.

2. 5 µl of a mixture of PEDOT:PSS and BSA (1:1), 5 µl of cell suspension, 5 µl of BSA, 5 µl of PBS were mixed and then 5 µl of this mixture was applied to the electrode.

3. A mixture of PEDOT:PSS and BSA polymer (7% aqueous solution) in a 1:1 volume ratio was applied to the working electrode in an amount of 5 µl, air-dried for 1 hour at room temperature, and then at + 4°C for 12 hours. 2.5 µl of cell suspension, 2.5 µl of BSA and 5 µl of PBS were mixed, and 5 µl of this mixture was applied to the electrode.

4. 5 µl of a mixture of PEDOT:PSS and BSA (1:1), 5 µl of a cell suspension, 5 µl of BSA, 5 µl of glutaraldehyde (2.5%) were mixed and apply 5 µl of this mixture to the electrode.

5. A mixture of PEDOT:PSS and BSA polymer (7% aqueous solution) in a volume ratio of 1:1 was applied to the working electrode in an amount of 5 µl, air-dried for 1 hour at room temperature, and then at + 4°C for 12 hours. 2.5 µl of cell suspension, 2.5 µl of BSA, 2.5 µl of glutaraldehyde (2.5%) and 2.5 µl of PBS were mixed, and then 5 µl of this mixture was applied to the electrode.

6. 5 µl of a mixture of PEDOT:PSS and BSA (1:1), 5 µl of a cell suspension, 5 µl of chitosan (2% solution in 1% acetic acid), 5 µl of PBS were mixed and then 5 µl of this mixture was applied to the electrode.

7. A mixture of PEDOT:PSS and BSA polymer (7% aqueous solution) in a volume ratio of 1:1 was applied to the working electrode in an amount of 5 µl, air-dried for 1 hour at room temperature, and then at + 4°C for 12 hours. 2.5 µl of cell suspension, 2.5 µl of chitosan (2% solution in 1% acetic acid), 5 µl of PBS were mixed, and 5 µl of this mixture was applied to the electrode.

8. 5 µl of a mixture of PEDOT:PSS and BSA (1:1), 5 µl of a cell suspension, 2 µl of Nafion, 8 µl of PBS were mixed and 5 µl of this mixture was applied to the electrode.

The resulting electrodes were dried at room temperature for 1 hour, and the response of the biosensors to the introduction of the same concentration of substrate (glucose, 3 mM) was measured.

3. Results

Figure S1 presents average signals for biosensors based on the eight compositions studied. From the presented data it is clear that the highest level of response to the introduction of glucose was observed for composition 1, in which cells were immobilized on the surface of a PEDOT:BSA mixture using Nafion gel. This effect can be attributed to the fact that Nafion protects cells from the potential negative effects of PEDOT:PSS, without interfering with electron transfer in the system. Figure S3 shows that in the case of using a mixture of components, the bacterial cells are separate

from each other, which apparently affects their ability to efficiently transfer electrons to the electrode. At the same time, when the layer-by-layer method is used, dense biofilm layers are formed, which leads to an increase in the electron transfer rate and results in an increase in the generated current. Separately, it should be noted that in compositions 4 and 5 the signal for the introduction of glucose is practically absent. This is due to the use of glutaraldehyde, which apparently has a significant toxic effect on *Gluconobacter* cells and almost completely inhibits their electrochemical activity.

The elemental content of the compositions used was studied with the method of EDX. EDX-SEM studies were carried out using an Oxford Instruments X-max 80 EDS system at an accelerating voltage of 10 kV. It is shown that the original PEDOT:PSS polymer is characterized by high sulfur content, the BSA solution - by high Si content, and fluorine also appears in the final composite due to the use of the sulfonated tetrafluoroethylene based fluoropolymer-copolymer Nafion.

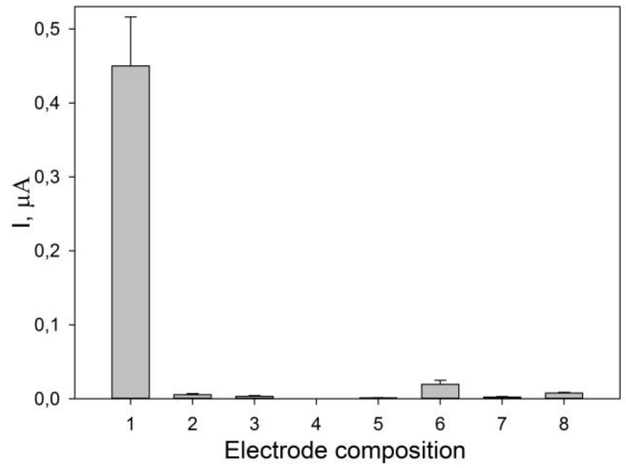

Figure S1. Dependence of biosensor signals on the composition of the working electrode.

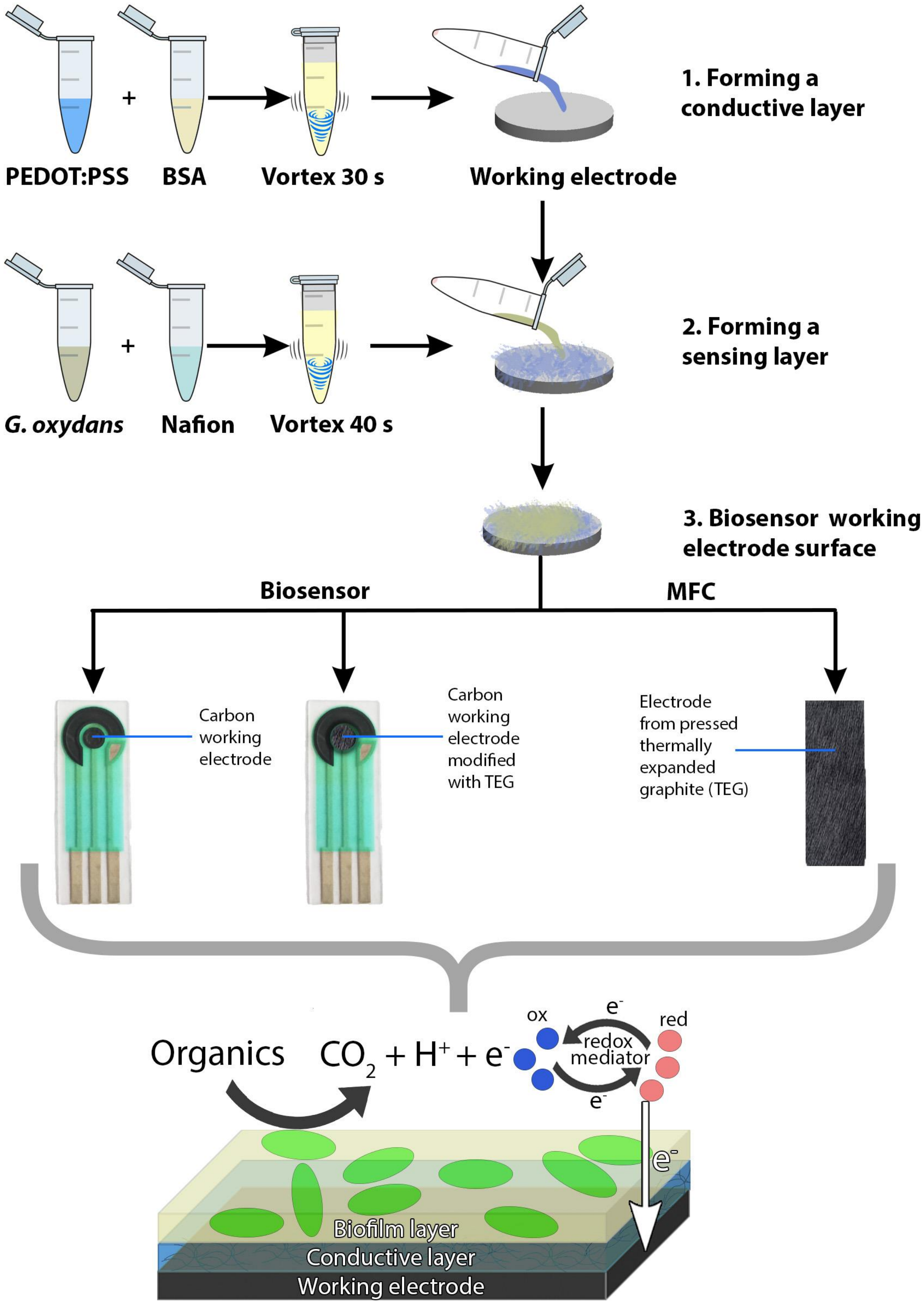

Figure S2. A schematic diagram of bioelectrode preparation and electron transport in system

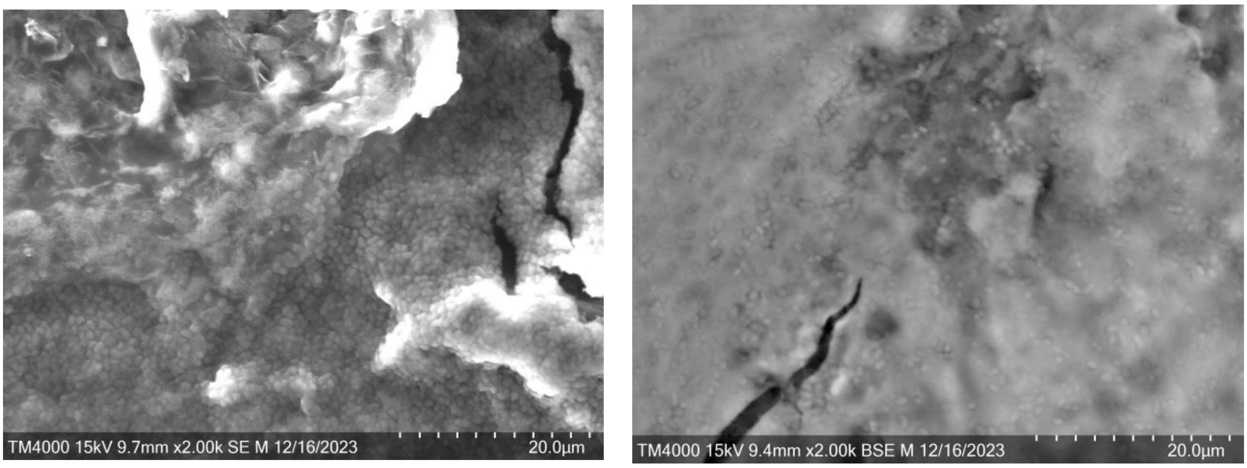

(**a**) (**b**)

Figure S3. Scanning electron micrographs of the surface of a graphite electrode covered with composition no. 1 (a) and composition no. 8 (b)

Table S1. Elemental composition of bioreceptor components.

	PEDOT:PSS		BSA		PEDOT:PSS/BSA		PEDOT:PSS/BSA/ *G. oxydans* /Nafion	
**Element**	**Weight %**	**Atomic %**	**Weight %**	**Atomic %**	**Weight %**	**Atomic %**	**Weight %**	**Atomic %**
C	47.40	62.85	50.65	67.16	50.11	63.31	59.52	68.86
O	24.89	24.78	9.56	9.52	25.69	24.37	20.90	18.15
Na	2.37	1.64	2.15	1.49	2.77	1.83	0.38	0.23
S	16.63	8.26	7.17	3.56	15.74	7.45	0.90	0.39
Cl	0.06	0.03	-	-	0.39	0.17	5.64	2.21
N	2.04	2.32	2.39	2.72	2.49	2.70	6.28	6.23
Si	0.19	0.09	28.08	13.30	0.19	0.11	0.71	0.35
F	-	-	-	-	-	-	3.75	2.74

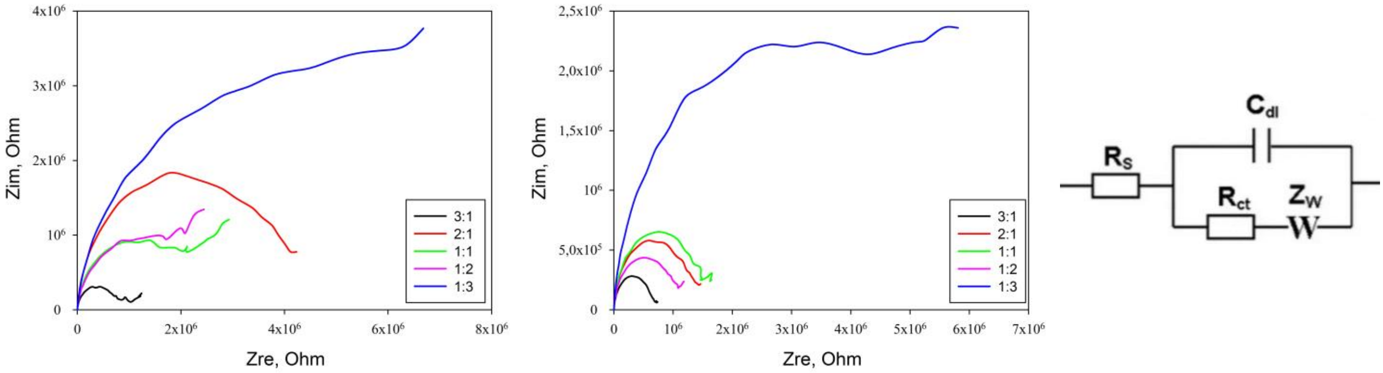

(**a**) (**b**) (**c**)

Figure S4. Nyquist diagrams for electrodes with different compositions of the working electrode: (a) in the absence of glucose, (b) in the presence of 3 mM glucose. The ratio of PEDOT:PSS and BSA is shown. (c) - Randles scheme; Rs – electrolyte resistance, C – electrode capacity, Rct – electrode charge transfer resistance, Zw – Warburg element

The influence of redox mediators on the respiratory activity of cells

The influence of mediators on the respiratory activity of cells in the presence of glucose was studied. A Clark-type oxygen electrode was used to record the rate of change in oxygen concentration upon addition of a substrate (glucose, 0.5 mM) in the presence and absence of a mediator. The tendency of the current level to zero corresponds to a decrease in the oxygen level in the system. Figure S5A shows typical biosensor signals for the introduction of glucose and HCF, and Figure S5B shows the biosensor signals for the introduction of glucose and 2,6-DCPIP.

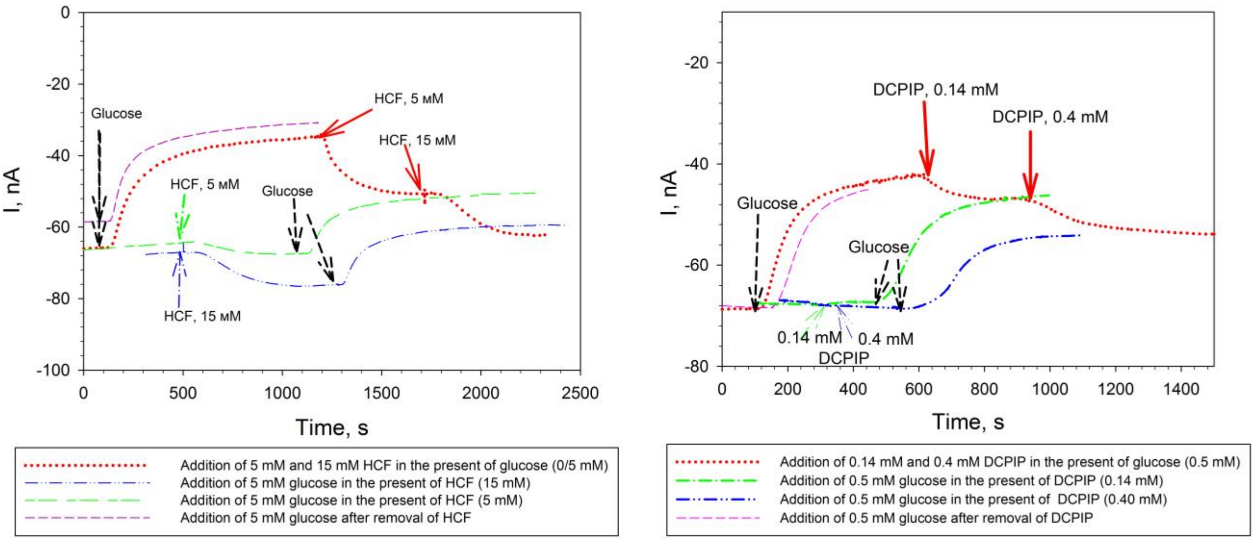

(**a**) (**b**)

Figure S5. The influence of electron transport mediators on the respiratory activity of cells in the presence of a substrate (glucose, 0.5 mM). (a) with the addition of HCF and (b) 2,6-DCPIP.

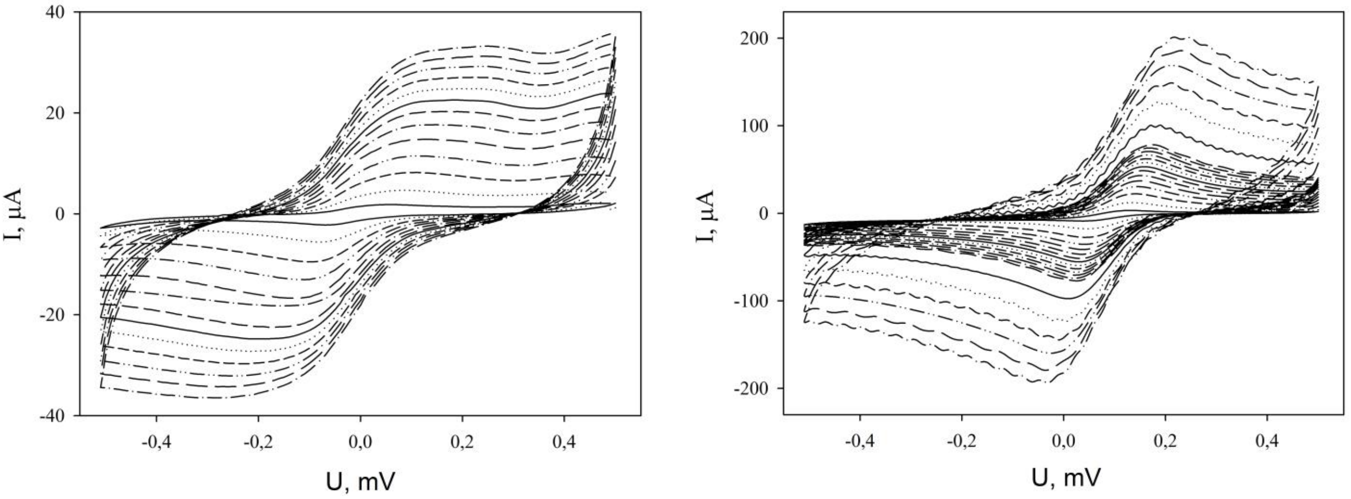

(**a1**) (**a2**)

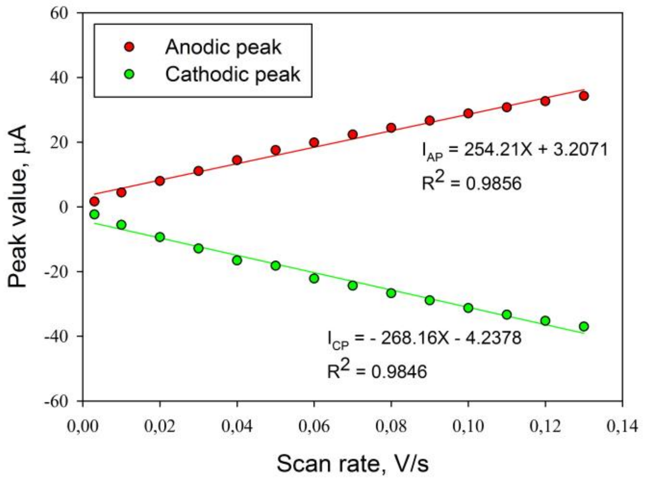

(**b1**)

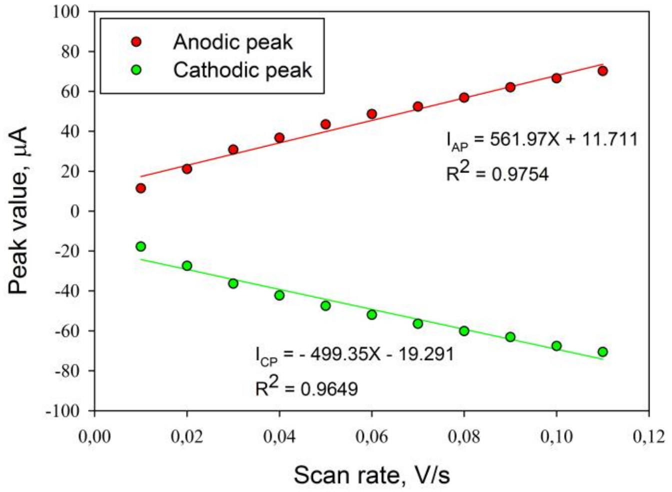

(**b2**)

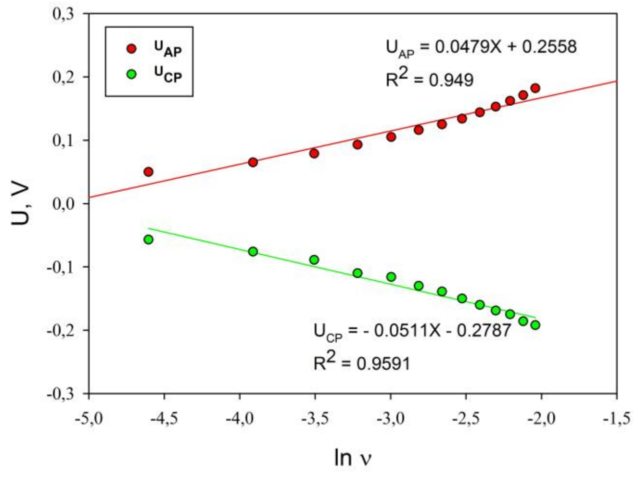

(**c1**)

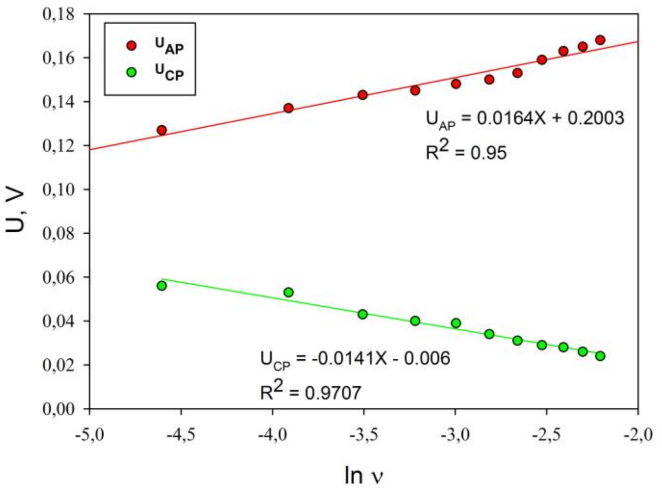

(**c2**)

Figure S6. Electrochemical characteristics of the electrode based on the PEDOT:PSS/BSA/*G. oxydans*/Nafion composition in the presence of redox mediators: 1 – DCPIP; 2 – HCF. (a) – cyclic voltammograms of electrodes at different scan rates; (b) – linear dependences of the anodic and cathodic peak currents on the scanning speed (I_{pa} – the magnitude of the anodic peak, I_{pc} – the magnitude of the cathodic peak); (c) - linear dependences of the anodic and cathodic peak potentials on the natural logarithm of the scanning speed (E_{pa} – anodic peak potential, E_{pc} – cathodic peak potential).

Determination of operational and long-term stability

To determine operational and long-term stability, a series of electrodes was prepared based on the most effective PEDOT:PSS/BSA ratio (1:1 v/v). For this ratio, the operational stability was checked, which was judged by the nature of the signal drop after the first measurement. The drop was 21% for the newly created biosensor. After the initial measurement and a rather significant drop, by the 3-5th measurement the signal stabilized and subsequently the drop in the biosensor signal did not exceed 10% for 10 measurements. The data is shown in Fig. S7. The long-term stability of the electrode is presented in Figure S8. It is shown that the electrode signal decreased by 77% during 140 days of electrode storage at +4 °C

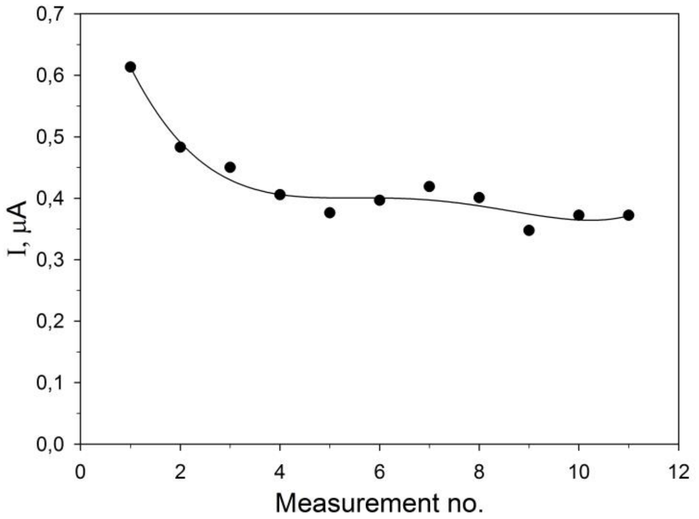

Figure S7. Operational stability of the electrode based on the conductive matrix PEDOT:BSA (1:1).

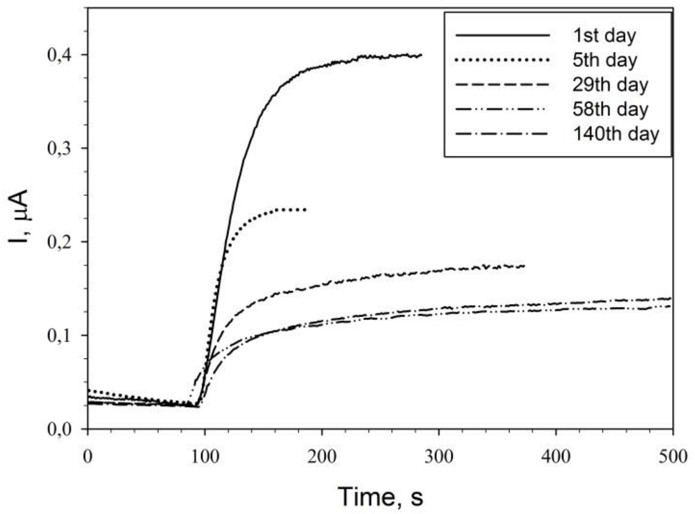

Figure S8. Changes in biosensor signals during storage for 4,5 months. The dry biosensor was stored at +4 °C between measurements.

Disclaimer/Publisher's Note: The statements, opinions and data contained in all publications are solely those of the individual author(s) and contributor(s) and not of MDPI and/or the editor(s). MDPI and/or the editor(s) disclaim responsibility for any injury to people or property resulting from any ideas, methods, instructions or products referred to in the content.
